# pHlash: A New Genetically Encoded and Ratiometric Luminescence Sensor of Intracellular pH

**DOI:** 10.1371/journal.pone.0043072

**Published:** 2012-08-14

**Authors:** Yunfei Zhang, Qiguang Xie, J. Brian Robertson, Carl Hirschie Johnson

**Affiliations:** Departments of Biological Science and Molecular Physiology and Biophysics, Vanderbilt University, Nashville, Tennessee, United States of America; Universidad de Castilla-La Mancha, Spain

## Abstract

We report the development of a genetically encodable and ratiometic pH probe named “pHlash” that utilizes Bioluminescence Resonance Energy Transfer (BRET) rather than fluorescence excitation. The pHlash sensor–composed of a donor luciferase that is genetically fused to a Venus fluorophore–exhibits pH dependence of its spectral emission *in vitro*. When expressed in either yeast or mammalian cells, pHlash reports basal pH and cytosolic acidification *in vivo*. Its spectral ratio response is H^+^ specific; neither Ca^++^, Mg^++^, Na^+^, nor K^+^ changes the spectral form of its luminescence emission. Moreover, it can be used to image pH in single cells. This is the first BRET-based sensor of H^+^ ions, and it should allow the approximation of pH in cytosolic and organellar compartments in applications where current pH probes are inadequate.

## Introduction

Intracellular pH regulation is vitally important for proper cellular function not only because virtually all enzymatic reactions are pH sensitive, but also because differences in pH across membranes allow compartmentalization of function (e.g., acidic lysosomes) and provide electrochemical gradients (e.g., to generate ATP). To maintain homeostatically optimum pH levels, cells constantly strive to offset acidic products of metabolism and mitigate influences of weak acids and bases in their environment by using an array of physiological buffers, proton pumps/channels, and ion transporters/antiporters that move H^+^ and H^+^-equivalent species across cellular membranes. Furthermore, changes of intracellular pH serve as modes of cellular regulation and signaling in a diverse collection of organisms and tissues. For example, pH changes activate gametes [1]–[3], regulate cell cycle progression [4], propagate apoptotic signals within cells [5], mediate cell elongation for plant gravitropism and root hair development [6], [7], accompany exocytosis [8]–[10], and associate with metabolic oscillations in yeast [11]. Acidification of the cytoplasm and the extracellular environment is linked with important physiological and pathological conditions, such as intense exercise, hypoxia and tumorigenesis [12]. Additionally, protons are known to compete for many of the same binding sites as other signaling ions (esp. Ca^++^ [13]), implicating H^+^ flux as a potential regulatory or cooperating factor in these ionic signaling pathways.

Identifying the prevailing influences of these co-occurring ion fluxes or correlating pH_i_ changes with cellular events requires methods for measuring intracellular pH. Classical methods included weak acid/base equilibrations or technically demanding microelectrode technology [14], but practically all current studies rely upon fluorescent indicators of pH [15]. These include membrane permeable fluorescent dyes such as BCECF (2′7′-bis-2-carboxyethyl-5- (and -6)-carboxyfluorescein) and SNARF (seminaphthorhodafluor) [15], which are popular because of their convenience and accuracy of pH estimation. In particular, these probes allow ratiometric measurements of pH_i_ so that variable uptake of the dyes and loss of fluorescence intensity due to photobleaching can be corrected (the ratiometry for BCECF is EX 440∶490 nm for an EM at 535 nm; the ratiometry for SNARF is EM at 580∶640 nm in response to EX at 488 nm). Despite their convenience, however, the fluorescent dyes cannot easily be targeted to specific subcellular compartments and they tend to become compartmentalized in some cell types over time [15]. Fluorescent pH-sensitive GFPs (e.g., “pHluorin” or “Pt-GFP” [8], [16], [17]) are particularly useful for approximating pH_i_ because they overcome some of the limitations of the fluorescent dyes. In particular, pH-sensitive GFPs can be genetically targeted to specific cell/tissue types and/or to specific subcellular compartments. Nevertheless, fluorescent pH sensors–be they dyes or pH-sensitive GFPs–suffer from the standard problems inherent in fluorescence excitation: photobleaching, poor penetration of tissue, and high background due to autofluorescence of cells/tissues. Finally, fluorescent reporters are difficult to use when excitation light can elicit a relevant photoresponse, such as in the retina. Moreover, the exciting advent of optically stimulating neural activity and/or gene expression [18], [19], merits a pH reporter that does not require excitation by light; optimally the monitoring of ion fluxes (i.e., with the pH sensor) should not constrain the light-induced stimulation of ion fluxes.

Therefore, the next generation of genetically encodable, ratiometric pH probes would optimally avoid excitation by light, as incumbent with fluorescence-based methods. We describe here a pH sensor that incorporates all the advantages of the pHluorins while avoiding fluorescence excitation by utilizing Bioluminescence Resonance Energy Transfer (BRET) [20], [21]. Bioluminescence is an enzyme-catalyzed reaction in which a luciferin substrate is oxidized by oxygen. Therefore, the luciferase enzyme mediates a chemiluminescence reaction in which the energy released is used to produce an intermediate or product in an electronically excited state, P*, which then emits a photon. The emission does not come from or depend on light absorbed, as in fluorescence or phosphorescence, but the excited state produced is similar to that produced in fluorescence after the absorption of a photon by the ground state of the molecule concerned. BRET avoids the problems of fluorescence excitation by using a luciferase as the donor and a fluorophore as the acceptor of resonance energy transfer, thereby avoiding excitation by an external light source. When the luciferase and fluorophore are brought within a radius of ∼50****Å, bioluminescence energy emanating from the luciferase can be directly transferred to the acceptor fluorophore by resonance energy transfer so that the spectrum of emitted bioluminescence is altered [20]. Consequently, BRET signals and images are acquired in complete darkness without fluorescence excitation. BRET can be measured in populations of cells, in tissues, or by imaging single cells [20]–[22]. We report here a novel pH reporter based on BRET, named “pHlash” (pronounced “flash” as in a flash of bioluminescence).

## Results

**Figure 1:**
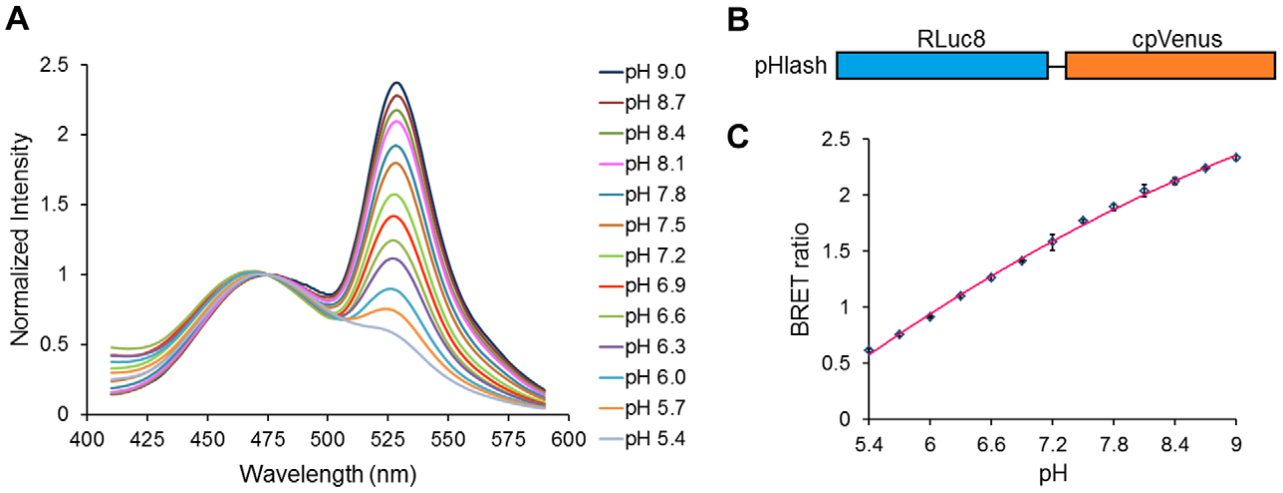
pH response of purified pHlash protein *in vitro*. (**A**) Normalized luminescence emission spectra of pHlash with 10 µM native coelenterazine at pH 5.4–9.0 (legend shown at right) of purified pHlash protein in 50 mM BIS-Tris-propane, 100 mM KCl, and 100 mM NaCl. Luminescence intensity was normalized to the peak at 475 nm (non-normalized data shown in Figure 2). (**B**) Construct of the pHlash fusion protein. Rluc8 was linked to cpVenus by the sequence Ala-Glu-Leu. (**C**) The BRET ratio (luminescence at 525 nm:475 nm) as a function of pH is shown for pHlash *in vitro*. Error bars are +/− S.D., but in most cases the error bars are so small that they are obscured by the symbols (n = 3).

**Figure 2:**
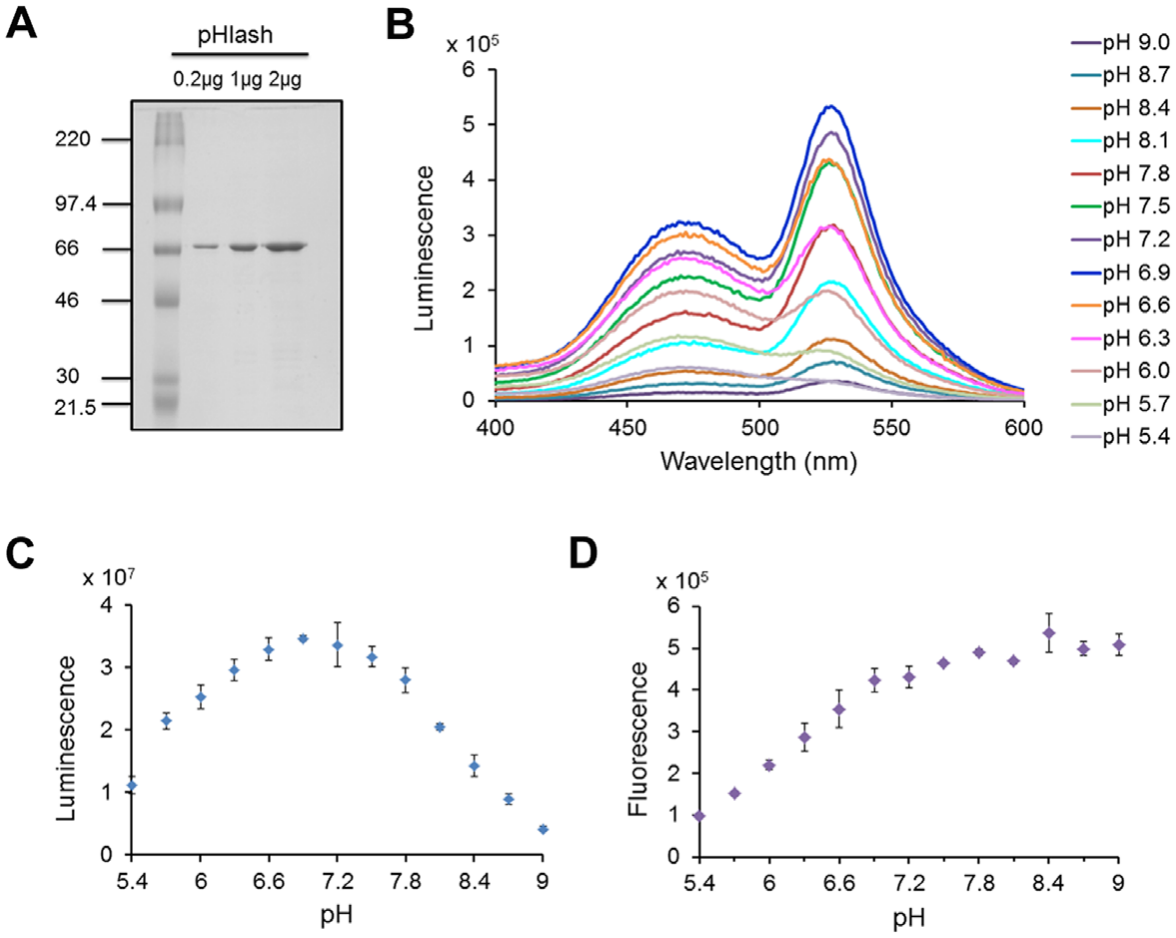
pH dependency of luminescence vs. fluorescence *in vitro*. (**A**) SDS-PAGE gel of purified His-tagged pHlash protein stained with Coomassie Blue dye. Leftmost lane is molecular weight standards with KDa indicated, while the other lanes are the purified pHlash protein loaded at 0.2, 1, and 2 µg per lane. (**B**) Raw data (not normalized) of luminescence emission spectra of purified pHlash protein at different pH values (pH 5.4–9.0), measured as in Figure 1. (**C**) pH dependence of total luminescence of pHlash (i.e., integration of total light emitted from 400–600 nm), (**D**) pH dependence of fluorescence emitted (510–600 nm) from pHlash during excitation at 490 nm (no substrate was presented to pHlash during the fluorescence measurements). Error bars are +/− S.D., but in some cases the error bars are so small that they are obscured by the symbols (n = 3).

**Figure 3:**
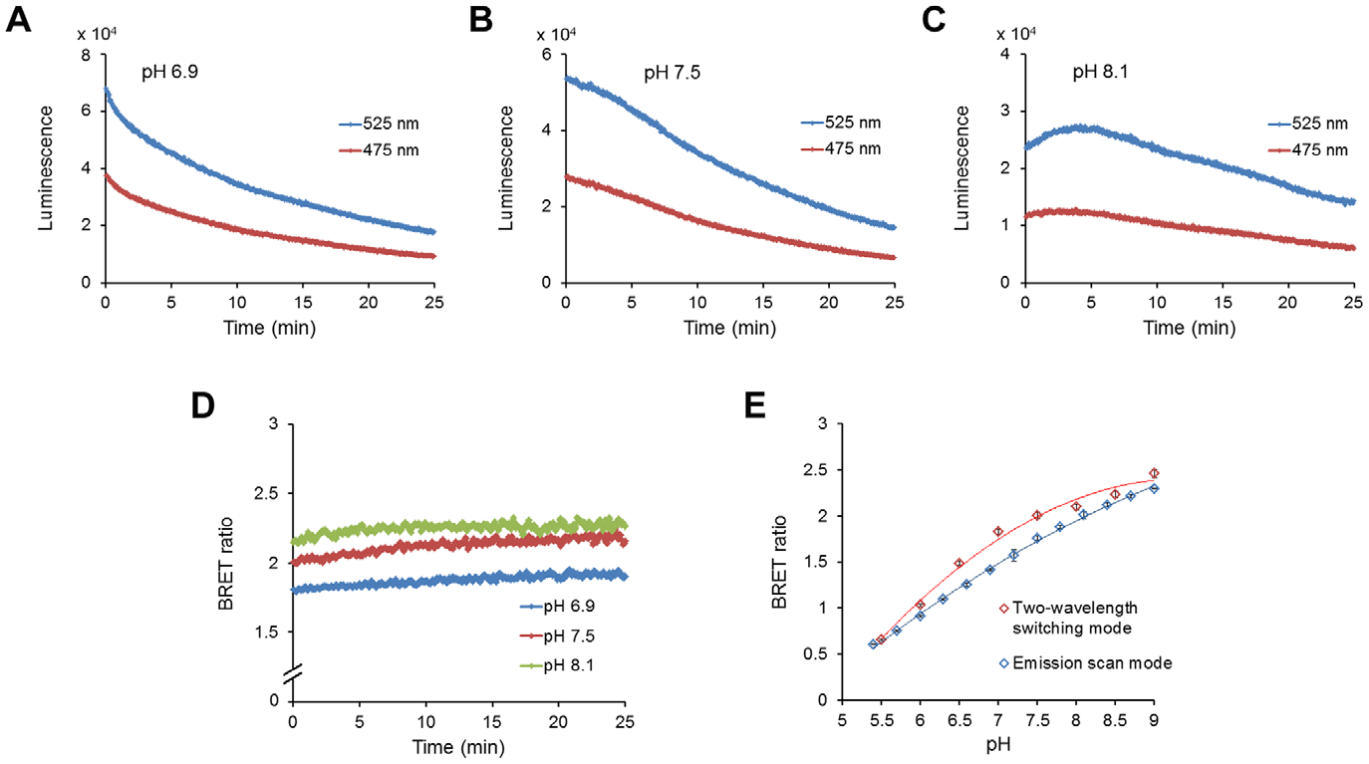
Time course recordings of purified pHlash protein at three different pHs *in vitro*. (**A**) pH 6.9, (**B**) pH 7.5, and (**C**) pH 8.1. (**D**) While the total luminescence intensity tends to decline over time after the addition of substrate, the BRET ratio (525∶475 nm) is constant over time. (**E**) Comparison of BRET ratio calibration measured *in vitro* by two different methods, the “Emission scan mode” and the “Two-Wavelength Switching Mode” (see Methods for details). Error bars are +/− S.D., but in most cases the error bars are so small that they are obscured by the symbols (n = 3).

To develop a genetically encodable and ratiometic pH probe that utilizes BRET rather than fluorescence excitation, donor luciferases were directly fused to acceptor fluorophores and assayed for pH dependence of resonance energy transfer. The BRET probe that we characterize here was the fusion of a bright mutant of *Renilla* luciferase (“Rluc8”) [23] through an Ala-Glu-Leu linker to the circularly permuted Venus fluorophore (“cpVenus”) [24], as shown in Figure 1. This fusion protein, called “pHlash,” shows a large change in its luminescence spectrum as a function of pH *in vitro* (Figure 1A shows the spectra normalized to the bioluminescence at 475 nm). When these data are plotted as a ratio of the emission at 525 nm to that at 475 nm, a clear pH dependency in the physiologically relevant range is obvious (Figure 1C). These spectra were measured *in vitro* with purified His-tagged pHlash protein (Figure 2A). When the spectral data are plotted without normalization (Figure 2B), it is clear that the light-emitting activity of Rluc8 in pHlash shows a pH dependency that is typical of enzymes, with peak activity at pH 6.9 (Figure 2C). Moreover, the fluorescence of the cpVenus moiety of pHlash also shows the well-characterized pH dependency of fluorescence emitted by GFP and its variants [16](Figure 2D). When the pH dependencies of the Rluc8 luminescence and the cpVenus fluorescence are taken into account, the pH dependency of pHlash’s spectra can be simulated on the basis of no pH dependency of the resonance transfer itself. The overall luminescence signal of pHlash when measured *in vitro* is not constant over time but decays significantly 20–30 minutes after the addition of the luciferin substrate for *Renilla* luciferase, coelenterazine (Figure 3A–C); however, the BRET ratio is constant (Figure 3D).

**Figure 4:**
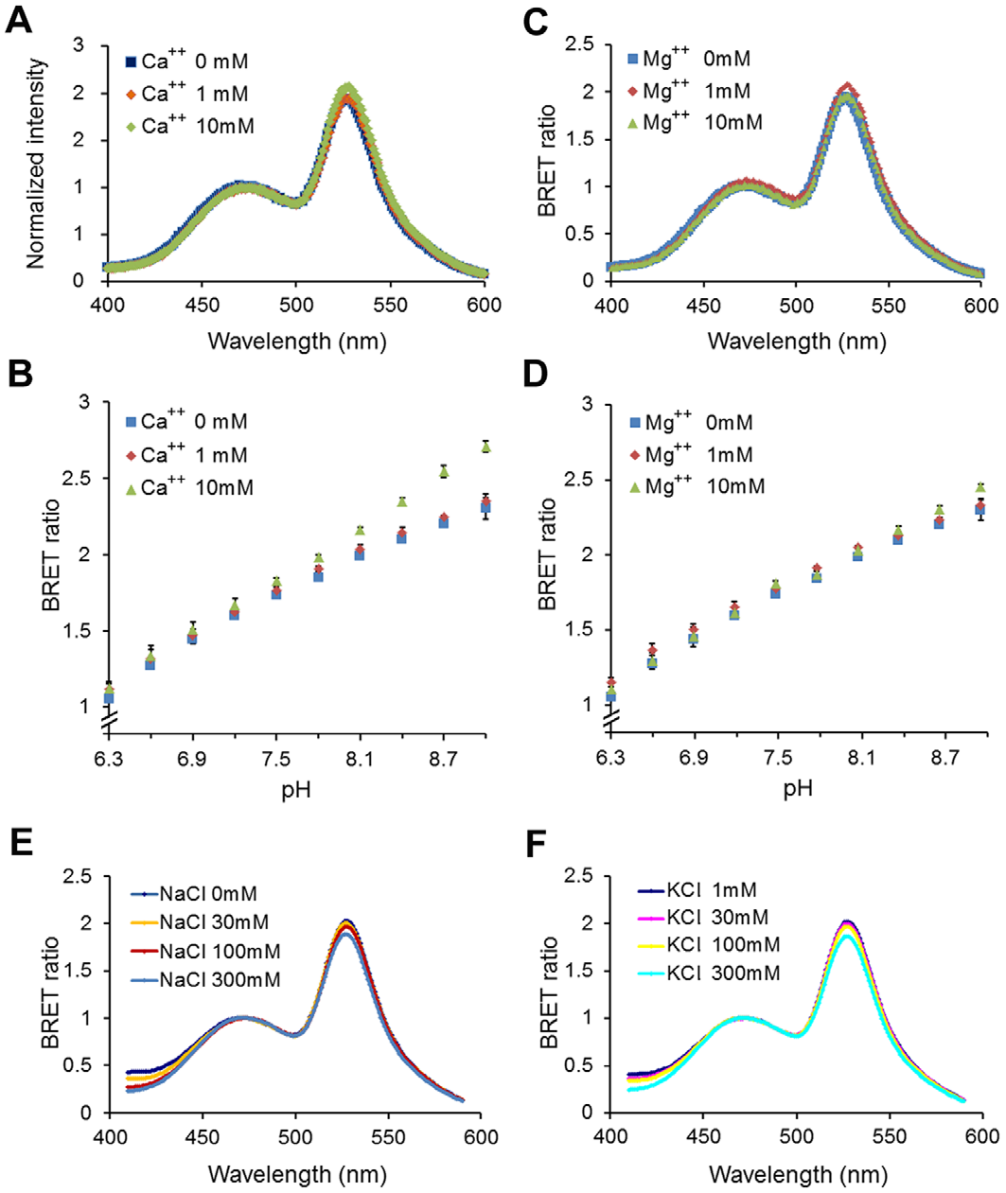
pHlash is not sensitive to other ions. (**A**) Normalized luminescence emission spectra at pH 7.5 in buffers with different amounts of added CaCl_2_. Spectra were normalized to the peak at 475 nm (there was a slight increase between 1 and 10 mM CaCl_2_ in the total luminescence intensity, but not in the 525∶475 nm ratio). (**B**) BRET ratio (525∶475 nm) at different [CaCl_2_]. Note that “0 mM CaCl_2_” means that no CaCl_2_ was added, and since there was ∼30 µM EGTA carried over from the enzyme stock solution, “0 mM CaCl_2_” will be sub-nanomolar concentrations of Ca^++^. (**C**) Normalized luminescence emission spectra at pH 7.5 in buffers with different amounts of added MgCl_2_. Spectra were normalized to the peak at 475 nm. (**D**) BRET ratio (525∶475 nm) of pHlash at different amounts of MgCl_2_. (**E**) Normalized luminescence emission spectra at pH 7.5 in buffers with different concentrations of NaCl. (**F**) Normalized luminescence emission spectra at pH 7.5 in buffers with different concentrations of KCl. The buffer used in these experiments was the same as described in Fig. 1 legend except with the addition of CaCl_2,_ MgCl_2_, NaCl, or KCl. For panels B and D, error bars are +/− S.D., but in most cases the error bars are so small that they are obscured by the symbols (n = 3).

To be an effective reporter of pH within cells, a sensor must be responsive to pH within the physiological range and that responsiveness must be specific for H^+^ so that it is not significantly affected by other common ions within cells. To identify a useful BRET reporter of intracellular pH, we tested three different candidate fusion proteins. In addition to the Rluc8/cpVenus fusion protein (pHlash) that is the topic of this paper, we also tested the previously reported eBAF-Y protein [25] and created a new fusion protein of *Gaussia* luciferase (“Gluc”) [26] with cpVenus (aka “hGluc-cpVenus”). Both eBAF-Y and hGluc-cpVenus showed excellent pH dependencies of their BRET ratio (Figure S2A,D). However, each had undesirable characteristics for use in cells; hGluc-cpVenus was secreted from cells even after Gluc’s putative secretion sequence had been removed, and eBAF-Y’s spectra were sensitive to varying salt concentrations that could interfere with its use *in vivo* (Figure S2E,F). In contrast, pHlash’s spectra were specific for pH. Neither CaCl_2_, MgCl_2_, NaCl, nor KCl (Figure 4) had a significant effect on the spectra or pH dependency of pHlash. Therefore, of the three potential BRET reporters of pH (pHlash, hGluc-cpVenus, and eBAF-Y) that we tested, only pHlash combined the desirable characteristics of sensitivity to pH, insensitivity to other ions, and retention within the cytoplasm. These characteristics suggested that pHlash could be an effective sensor of pH within cells.

**Figure 5:**
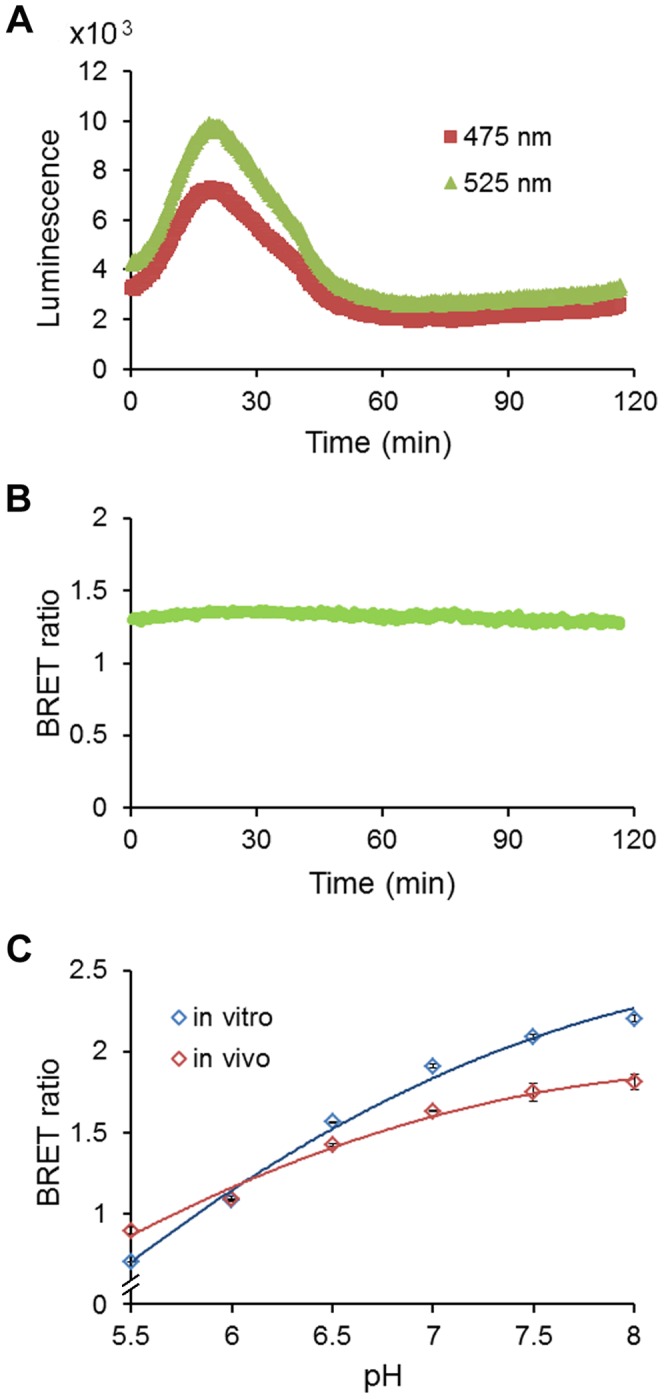
Expression of pHlash in yeast cells (strain CEN.PK113-7D). (**A**) A 120 min recording of yeast cells with 10 µM native coelenterazine (50 mM KCl, 50 mM NaCl, 50 mM HEPES buffer, pH 7.0). (**B**) Although the luminescence level increased then decreased gradually (as shown in panel A), the BRET ratio (525∶475 nm) was relatively constant over time after the addition of coelenterazine. (**C**) Comparison of pH response of yeast expressing pHlash in calibration buffer at various extracellular pHs (*in vivo*, blue symbols) with pHlash protein *in vitro* (red symbols, *in vitro* data from Figure 1). Error bars are +/− S.D., but in most cases the error bars are so small that they are obscured by the symbols (n = 3).

**Figure 6:**
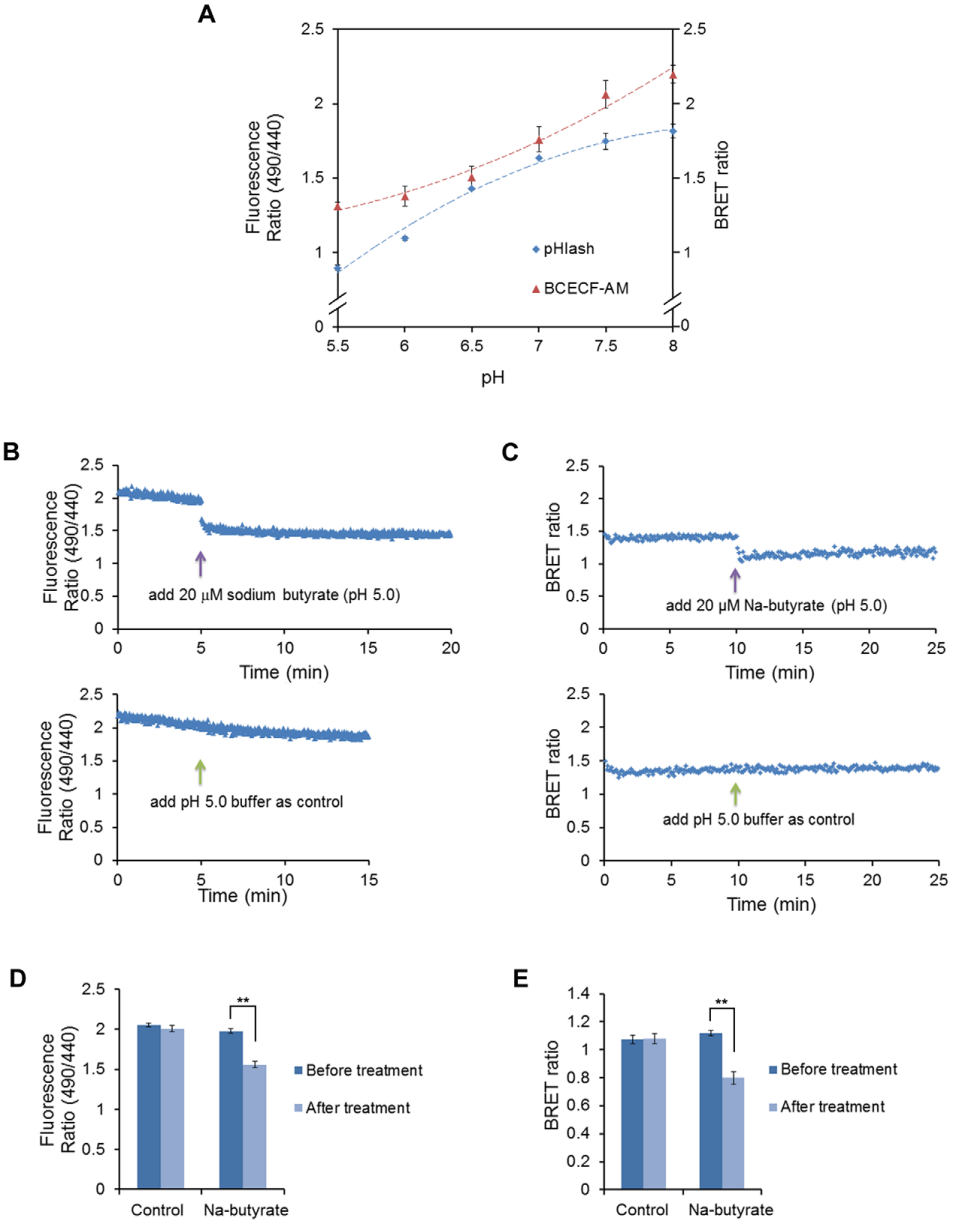
Response of yeast pH to weak acid treatment. (**A**) pH response of BCECF-AM loaded yeast cells (red) compared with pHlash transformed yeast cells (blue). (**B**) Response of BCECF-AM loaded yeast cells to weak acid (20 µM sodium butyrate, upper panel). Yeast cells were suspended in 20 mM MES buffer (pH 5.0). After 10 min baseline recording, 20 µM final concentration of Na-butyrate (pH 5.0) was added (total added volume was 20 µl, which is the same volume as that for the pH 5.0 buffer that was used as a control in the lower panel). (**C**) Response of pHlash-expressing yeast cells to weak acid. Yeast cells were suspended in 20 mM MES buffer (pH 5.0) with 10 µM native coelenterazine. After 10 min baseline recording, 20 µM final concentration of Na-butyrate (pH 5.0) was added (upper panel). An equal volume of pH 5.0 buffer was added as control (lower panel). The change in BRET ratio of pHlash reports the intracellular acidification after treatment with Na-butyrate. (**D**) Histogram depiction of multiple replicates of the BCECF protocol illustrated in panel B. (**E**) Histogram depiction of multiple replicates of the pHlash protocol illustrated in panel C. In panels A, D, and E, error bars are +/− S.D., but in some cases the error bars are so small that they are obscured by the symbols (n = 3 for panel A, n = 5 for panels D and E). ** p<0.01.

To test if pHlash could accurately report pH *in vivo*, we constructed a yeast strain in which pHlash was expressed in the cytosol. When native coelenterazine was added extracellularly, the total luminescence emission increased and then decreased (Figure 5A), but the BRET ratio remained constant for at least 120 min (Figure 5B). When yeast cells expressing pHlash were placed in a buffer that brings the cytosolic pH of yeast closer to the extracellular pH [27], the calibration of pHlash’s BRET ratio *in vivo* showed an excellent correspondence with its BRET ratio *in vitro* (Figure 5C). Using this calibration method, the BRET ratio of pHlash showed a similar dynamic range to that of the well-characterized BCECF method in yeast cells (Figure 6A). In addition, responses of BCECF to rapid acidification of cytosolic pH *in vivo* induced by the weak acid butyrate (Figure 6B) were similar to those measured by pHlash (Figure 6C); the ratio change of pHlash in response to acidification by 20 µM butyrate is at least as large as that of BCECF (Figure 6D,6E).

**Figure 7:**
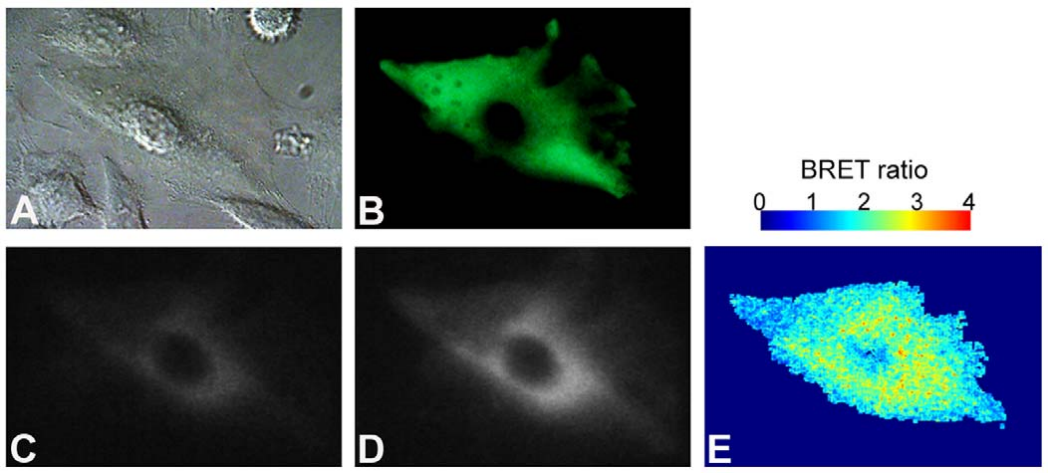
BRET imaging of pHlash-expressing HeLa cell. (**A**) Bright-field image of a HeLa cell in DMEM including 10% FBS by DIC (differential interference contrast). (**B**) Fluorescence of the cpVenus moiety of pHlash. (**C**,**D**) Dual-View™ image of BRET signals from the cell in 2.5 µM ViviRen™. Luminescence images were split at 505 nm by the Dual-View™ microimager into ∼400–505 nm wavelengths (panel C) versus ∼505–600 nm wavelengths (panel D). (**E**) Spatial distribution of BRET ratios (505–600 nm/400–505 nm) over the entire image (pseudocolor scale shown above panel E). Red dots are off-scale values. Imaging was performed as 10 sequential 2 sec exposures that were integrated by choosing the median value for each pixel over the sequence of 10 exposures (Apo N 60 × objective, NA 1.49). In this particular cell, pHlash appears to be excluded from the nucleus, but in other cells (e.g., see Figure 9), pHlash is present throughout the cell, including in the nucleus.

**Figure 8:**
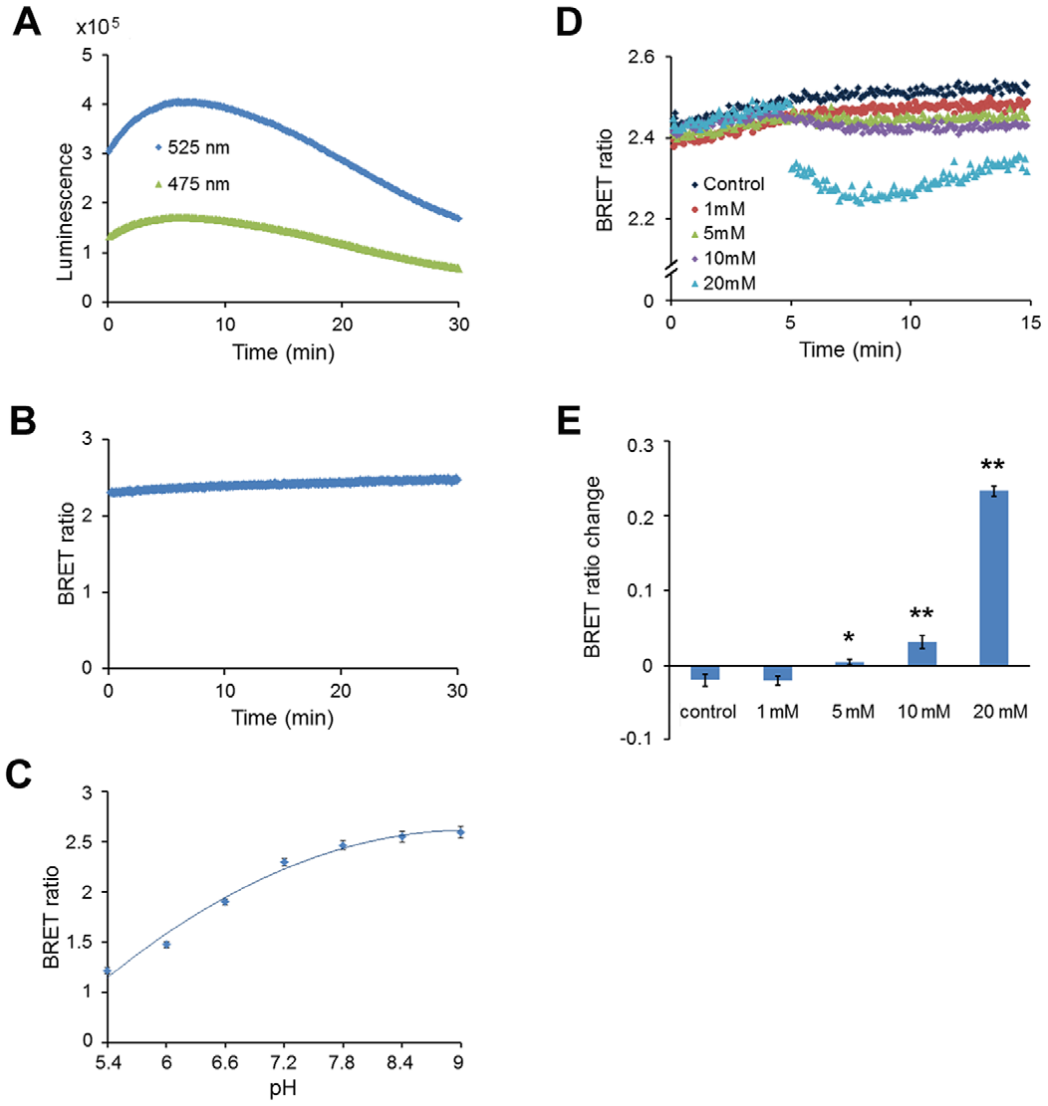
Response of pHlash to acidification of the cytoplasm in HeLa cells. (**A**) Time-course recording of total luminescence from pHlash-transfected HeLa cells with 2.5 µM ViviRen™ as substrate. Luminescent signals were recorded at 475 nm and 525 nm using the QM-7/SE spectrofluorometer. (**B**) Although the luminescence level increased then decreased gradually (as shown in panel B), the BRET ratio (525∶475 nm) was relatively constant over time after the addition of ViviRen. (**C**) Calibration of pH in nigericin-treated HeLa cells expressing pHlash with 2.5 µM ViviRen™ as substrate. Extracellular pH (pH 5.5∼9.0) was modified in the following medium: 20 µM nigericin, 100 mM KCl, 100 mM NaCl, 1.36 mM CaCl_2_, 4.5 g/l glucose, 50 mM BIS-Tris-propane (pH 5.5∼9.0). (**D**) Response to treatment with NaF at different concentrations (1 mM, 5 mM, 10 mM, or 20 mM). Transfected HeLa cells were recorded for 5 min with 2.5 µM ViviRen™ in the following medium: 150 mM NaCl, 5 mM KCl, 1.36 mM CaCl_2_, 4.5 g/l glucose, 50 mM BIS-Tris-propane (pH 7.4). Five min after the addition of ViviRen™, NaF (or 20 µl medium as control) was added to the indicated final concentrations. (**E**) Statistics of change in BRET ratio of HeLa cells with different concentrations of NaF. * p<0.05, ** p<0.01 as compared to the BRET ratio change of the controls. In panels C and E, error bars are +/− S.D., but in some cases in panel C the error bars are so small that they are obscured by the symbols (n = 3 for panel C, n = 5 for panel E).

We pursued a similar approach to ascertain if pHlash could be used to measure pH in mammalian cells. HeLa cells that were transfected with a construct to express pHlash in the cytosol exhibited levels of luminescence that could be imaged in single cells incubated in the serum-insensitive substrate ViviRen™ [22](Figure 7). A representative HeLa cell was viewed by DIC in Figure 7A and the fluorescence of the cpVenus moiety of pHlash was imaged in Figure 7B. A Dual-View™ microimager was used to simultaneously collect images from two wavelength ranges by including a dichroic that splits the image at 505 nm and short-pass/long-pass filters that refine the spectral distinctions (400–505 nm in Figure 7C and 505–600 nm in Figure 7D). The spatial distribution of BRET ratios calculated over this HeLa cell is shown in Figure 7E and ranged around 2.4 (∼pH 7.6 from the calibration curve in Figure 8C; pseudocolor scale is shown above panel E), as determined by a pixel by pixel comparison of panels C and D in Figure 7.

**Figure 9:**
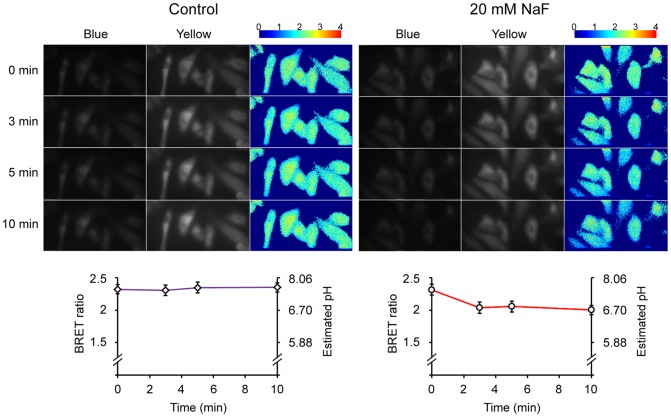
BRET imaging of pHlash-transfected HeLa cells after NaF treatment. Cells were imaged as in Figure 7 at different times after adding an equal volume of control medium or an equal volume of medium containing 40 mM NaF to achieve a final concentration of 20 mM NaF (both media contained 2.5 µM ViviRen™). The luminescence images from the blue (∼400–505 nm) and yellow (∼505–600 nm) channels of the Dual-View™ were the median values after 7 sequential 2-sec exposures. Cells were imaged at 0, 3, 5, and 10 min after adding control or NaF-containing media (time 0 =  image before adding media). Ratio images were made by the images obtained from the yellow channel divided by the images from the blue channel (505–600 nm/400–505 nm), and the scales illustrate the pseudocolor encoding of BRET ratio. The plots under the BRET images show the time-dependent changes in the calculated BRET ratio and in the cellular pH estimated from the *in vivo* calibration curve shown in Figure 8C; values are shown as mean ± S.D. (n = 6 cells for both the control and NaF sets).

HeLa cells in suspension as measured with the QM-7/SE spectrofluorometer exhibited a stable BRET ratio for at least 30 min (Figure 8B, even though the total luminescence increased and subsequently decreased at 475 nm and 525 nm as shown in Figure 8A) and a similar BRET ratio to that obtained with the Dual-View™ microimager (Figure 7E). Finally, when HeLa cells were induced to generate acid by the addition of sodium fluoride [28], the cytosolic pH as monitored by pHlash dropped (Figure 8D) with a concentration-dependent relationship (Figure 8E). After five min of treatment with 20 mM NaF, the BRET ratio begins to recover. This is the same time course of the pH change within cells treated with sodium fluoride, indicating that changes of intracellular pH have reversible effects upon the BRET ratio of pHlash. We also imaged this NaF-induced acidification from single cells, as shown in Figure 9. In cells treated with control medium, the pH estimated by pHlash remained relatively constant with an estimated pH of 7.4–7.5, while the pH estimated by pHlash in the NaF-treated cells dropped from pH 7.45 (at time 0) to pH 6.70 (at 10 min after the addition of 20 mM NaF).

## Discussion

We took advantage of BRET to develop a genetically encodable and ratiometic pH probe that could be ideal for applications where autofluorescence, tissue penetration, excitation-induced phototoxicity, and photoresponsiveness are undesirable. For example, green plant tissue is highly pigmented and strongly autofluorescent. While many studies using fluorescent probes have been successfully conducted in plants with judicious choices of filters, nevertheless a BRET-based probe should be superior for avoiding these problems of plant autofluorescence [21], [22]. Photoresponsive tissue is another application where a BRET-based reporter of pH could shine. The most obvious example is in the retina, where bioluminescence reporters have been used effectively to report calcium ion fluxes without stimulating retinal responses because the levels of luminescent light emission were very low [29]. However, many other cell types have photoresponses, including plant cells and many unicellular organisms, and reporters based on luciferase-catalyzed luminescence that can be used in complete darkness would be valuable in these applications. Another example of photoresponsiveness results from the innovative use of light-induced channels to optically excite neural activity and/or gene activity via channel-rhodopsins [18], [19]. A sensor that requires photonic excitation will likely stimulate the very process that the reporter is supposed to be measuring without perturbing. Genetically encoded sensors of pH have proven to be useful reporters of neural activity by sensing synaptic vesicle fusion [8], [10]. However, if the pH sensor requires excitation, it could directly stimulate channel-rhodopsins [18], [19], thereby perturbing the very process it is intended to monitor. In this application, a BRET-based reporter of pH could partner more effectively with optogenetic stimulation because it does not require excitation. Therefore, a BRET-based reporter could monitor pH in darkness, then a brief flash of light could be applied to stimulate the optogenetic probe, after which time the BRET reporter could be consulted to assess the cellular response.

The pHlash sensor shares advantages of optimal fluorescence-based probes. In particular, it is genetically encoded so that it should be possible to target pHlash to subcellular compartments to measure the pH within organelles and microdomains within cells. An advantage of choosing a luciferase that uses coelenterazine (or the serum-insensitive ViviRen™) as its substrate is that coelenterazine already has a well-characterized ability to penetrate throughout organelles and microdomains of cells and act as a substrate for luciferases, for example into the mitochondria of animal cells [30], synaptic termini of neurons [31], plasma membrane microdomains [32], and into the chloroplast and nuclei of plants [33], [34]. In addition to its ability to be targeted to microdomains, *Renilla* luciferase (the luciferase of pHlash) retains its activity when fused to other proteins [20]–[23], [32], [34]. Finally, pHlash is a ratiometric probe, so its signal (the BRET ratio) compensates for potentially varying levels of expression; one cell may express pHlash strongly and have a bright luminescence signal whereas another cell may have low expression and only dim luminescence–nevertheless the BRET ratio should be equivalent if the pH within these hypothetical cells is equal.

The BRET technology “tool-kit” has increased significantly [21], [22] since its first introduction [20]. Nevertheless, BRET measurements can be challenging. Its two major liabilities are the weakness of the luminescence level and the stability of the luciferase-catalyzed signal. The first “liability” is perhaps a blessing in disguise because it enables BRET-based probes to be used in photoresponsive tissue. Recent developments have improved the detection of dim signals: brighter luciferases [23], substrates such as ViviRen™ that are compatible with cells in complex medium [22], and more sensitive cameras [22]. The sensitivity of the cameras together with the dim signals require that background light be absolutely excluded for BRET measurements/imaging. Another characteristic of BRET measurements *in vivo* that can be a liability in some cell types and/or media is that the overall luminescence output can sometimes change over time (usually an increase followed by a decline, as in Figures 5A for yeast cells and 8A for HeLa cells). For mammalian cell cultures in serum-containing medium (and possibly for other types of cells in complex medium), ViviRen™ gives signals that have a usable lifetime of at least 30 minutes (Figure 8B) [22]. A 30-min time frame is usually sufficient for a screening assay in plates or a microscopic measurement. In the *in vitro* assay, the signal also decays over 30 min (Figure 3). For plant cells in simple salt medium, however, *Renilla* luciferase emits stable signals lasting >2 hours using native coelenterazine [22]. The reasons for these differences in stability are not known, but improved stabilization of signals over longer time intervals would make coelenterazine-based luminescence methods even more useful. Finally, the *in vivo* calibration curves for pHlash (Figures 5C, 6A, and 8C) show larger incremental changes in BRET ratio for pH changes that are more acidic than the typical cytosolic pH of 7.0–7.4 than for pH values that are more alkaline; consequently, when pHlash is expressed in the cytosolic compartment, it may be a more useful reporter for acidifications of the cytosol (as depicted in Figures 6C, 8D, and 9) than for alkalinizations. Nevertheless, pHlash combines many desirable characteristics for reporting intracellular pH and heralds the possibility of other BRET-based ion/molecule sensors.

## Materials and Methods

### Genetic Constructs

The pHlash construct encodes a fusion protein of Rluc8 [23] and cpVenus (cp173Venus) [24] linked by the sequence GCCGAGCTC encoding the amino acids Ala-Glu-Leu. The hGcpV construct encodes a fusion protein of humanized *Gaussia* luciferase (hGluc) [26] and cpVenus [24] linked by the sequence GCCGCCCGC encoding the amino acids Ala-Ala-Arg. The eBAF-Y construct encodes a fusion protein of EYFP and Rluc8 [25]. Plasmids were used for protein expression in *E. coli* (pRSETb from Invitrogen), in yeast cells (*Saccharomyces cerevisiae* strain CEN.PK113-7D; pRS305 [29]), and in HeLa cells (pcDNA3.1+ from Invitrogen).

### Protein Expression and Purification

For the expression of His-tagged fusion proteins (on the N-terminus) of pHlash, hGluc-cpVenus, and eBAF-Y, *E. coli* strain BL21 (DE3) cells bearing pRSETb harboring the fusion sequence were grown overnight in 10 ml LB medium with ampicillin (60 µg/ml) at 37°C, after which time the cultures were diluted into 500 ml fresh medium and grown until OD_600_ reached 0.6. The expression of fusion proteins was induced by 1 mM isopropyl-β-D-thiogalactopyranoside (IPTG). After incubation with IPTG for 5 h at 25°C, cells were harvested by centrifugation, suspended in 25 ml buffer (50 mM sodium phosphate, 300 mM NaCl, pH 7.0), and disrupted by sonication. After the cell debris was removed by centrifugation, the fusion proteins were purified on TALON metal affinity resin according to the manufacturer’s protocol (Clontech)(Figure 2A). Eluted pHlash protein was suspended in 30 mM MOPS buffer (pH 7.2), quantified using the Bio-Rad protein assay, flash-frozen with liquid nitrogen, and stored at –80°C for later use.

### *In vitro* pH Calibration

*In vitro* pH calibration was performed in 50 mM BIS-Tris-propane, 10 mM KCl, and 100 mM NaCl, that had been adjusted with 1N HCl to 13 different pH values ranging from 5.4 to 9.0 (this is “calibration buffer”). Purified pHlash protein (1 µg in 2 µl of the MOPS storage buffer) was diluted 250X to achieve a final concentration of 1 µg purified protein per 500 µl calibration buffer for each individual measurement. Because “0 mM CaCl_2_” means that no CaCl_2_ was added, and since there was ∼30 µM EGTA carried over from the enzyme stock solution, “0 mM CaCl_2_” will achieve sub-nanomolar concentrations of Ca^++^. Calibration curves were derived from measurements of the BRET by two different methods: (1) Emission Scan Mode in the QM-7/SE spectrofluorometer, where the spectrum was continuously scanned from 400–600 nm as in Figures 1A, 2B, 4, S1, and S2 (this required about 24 sec for an entire spectral scan from 400–600 nm), or in Two-Wavelength Switching Mode in the QM-7/SE spectrofluorometer, where the monitoring of luminescence emission was alternately switched between 475 nm and 525 nm for measurement of time courses as in Figures 3, 5, 6, and 8 (i.e., 0.1 sec at 475 nm followed by 0.1 sec at 525 nm and repeated continuously, but the switching requires additional time, so the average time for one cycle to measure luminescence at both 475 nm and 525 nm is about 1.5 sec). Approximately 20 cycles of 475 nm/525 nm switching was performed and the BRET ratios averaged for each datum plotted in Figures 3, 5, 6, and 8. The calibration curve obtained with the Emission Scan Mode is more linear than that obtained with the Two-Wavelength Switching Mode (Figure 3E). However, the Two-Wavelength Switching Mode was needed for the time-course measurements of Figures 3, 5, 6, and 8.

### BRET Measurement

Substrates for luciferase were native coelenterazine (NanoLight, Pinetop, AZ) or ViviRen™ (a serum-insensitive version of coelenterazine-h; Promega, Madison WI) at a concentration of 10 µM (or as indicated). For measurement of BRET emission (except the imaging measurements–see below), a QuantaMaster QM-7/SE (Photon Technology International, Birmingham NJ) spectrofluorometer was used. For luminescence spectral measurement, the excitation beam was shut off, and the slit width was set to 16 nm. For live cell measurement, a stir bar was placed on the bottom of the cuvette for gentle stirring to maintain the cells in suspension. The units of luminescence measurement are counts per second (cps).

### *In vivo* pH Calibration of Yeast Strains Expressing pHlash

To construct yeast cells that express pHlash protein, the coding sequence of pHlash was transferred to a yeast expression plasmid that had been constructed from the pRS305 backbone [35] with an added *hphNT1* hygromycin resistance gene from pYM-24 [36]. The *ACT1* promoter from *S. cerevisiae* was placed immediately upstream of pHlash and the *ADH1* terminator from *S. cerevisiae* was placed immediately downstream. The pHlash expression plasmid was stably integrated into the genome of *S. cerevisiae* strain CEN.PK113-7D through homologous recombination with the endogenous *LEU2* gene.

*In vivo* pH calibration was performed in yeast cells expressing pHlash that were made slightly porous to protons by incubation in the following yeast permeabilization buffer: 50 mM MES, 50 mM HEPES, 50 mM KCl, 50 mM NaCl, 0.2 M ammonium acetate, 10 mM NaN_3_, 10 mM 2-deoxyglucose, 75 µM monensin, and 10 µM nigericin, titrated to 8 different pH values adjusted with 1 M NaOH from 5.5 to 9.0 (methodology described in [27]). BCECF-AM (Molecular Probes, Eugene OR) was used as a control for pHlash in the pH calibration in permeabilized yeast. For *in vivo* calibration using BCECF, yeast cultures were incubated in YPD medium (at pH 7.5) with 50 µM BCECF-AM at 30°C for 30 min, washed and suspended in yeast permeabilization buffer as described in [27]. Dual excitation at 440 nm and 490 nm was used; emission at 535 nm was recorded with the QuantaMaster QM-7/SE spectrophotometer.

### Mammalian Cell Culture and Transfection

HeLa cells (obtained from ATCC, ATCC® Number: CCL-2™) were grown in DMEM medium (Invitrogen) with 10% FBS at 37°C with 5% CO_2_ and transfected with pcDNA3.1+ harboring the pHlash sequence under the control of the CMV promoter using FuGene6 (Invitrogen, Carlsbad, CA) according to the manufacturer’s instructions. After 24 h, cells for imaging or BRET measurement were washed and resuspended in either (1) DMEM medium without phenol red +10% FBS, or (2) 150 mM NaCl, 5 mM KCl, 1.36 mM CaCl_2_, 4.5 g/l glucose, 50 mM BIS-Tris-propane (pH 7.4). For calibration of pH in HeLa cells expressing pHlash as in Figure 8C, extracellular pH was modified over a range of 5.5 to 9.0 in the following medium: 100 mM KCl, 100 mM NaCl, 1.36 mM CaCl_2_, 4.5 g/l glucose, 20 µM nigericin, and 50 mM BIS-Tris-propane (pH 5.5∼9.0).

### Imaging of BRET from HeLa Cells

BRET imaging was accomplished using (i) a Dual-View™ micro-imager and (ii) a modified electron bombardment-charge coupled device (EB-CCD) camera as described previously [22]. The Dual-View™ micro-imager (Optical Insights, Tucson AZ, USA) allows the simultaneous acquisition of luminescence images at two wavelengths; therefore, a “BRET ratio” of emission in the two ranges can be calculated without the complication that the total luminescence signal may be changing over the time course of the exposure. The Dual-View™ consists of a dichroic mirror (in our case, to split at 505 nm using Q505LPxr) and interference filters to select for wavelengths below 505 nm (HQ505SP) and for wavelengths above 505 nm (HQ505LP). Our EB-CCD camera had a GaAsP photocathode with low ion feedback and cooling to −25°C (Hamamatsu Photonic Systems, Bridgewater NJ, USA). This EB-CCD camera model C7190-13W has a resolution of 512 X 512 pixels with a pixel size of 24 X 24 µm. The acquisition software was Photonics-WASABI (Hamamatsu). The Dual-View™ and EB-CCD were attached to the bottom port of an IX-71 inverted microscope (Olympus America Inc., Melville NY, USA). This setup allows the measurement of fluorescence using an epifluorescence attachment (EX 500/20 nm, EM 520 LP). The entire IX-71 microscope was enclosed in a temperature controlled (22–37°C) light-tight box. The IX-71 microscope was used with an Apo N 60 × objective, NA 1.49 (oil immersion, Olympus). For single HeLa cells, 10 sequential 2 sec exposures were integrated by choosing the median value for each pixel over the sequence of 10 exposures. Then background subtraction was performed with ImageJ by using a single pixel from the non-sample region of the image as a background value for both plant and mammalian samples. A pixel-by-pixel BRET ratio was calculated with ImageJ, and the numerical ratios were visualized with a pseudocolor look-up-table (LUT) as displayed above Figure 7E. For imaging DIC and cpVenus fluorescence (as in Figures 7A and 7B), a color DP72 camera (Olympus) was used from the side port of the IX-71 microscope.

## Supporting Information

Figure S1 **Data for some of the pH values shown in** **Figure 2B** **replotted as a single trace per pH assessment of the BRET emission spectrum.** Representative pHs throughout the entire pH range are shown. Data are not normalized, note that the values of the ordinates are different among the various plots.
(TIF)

Figure S2 **Response of purified hGluc-cpVenus and eBAF-Y** **to pH and NaCl/KCl.** (**A**) purified hGluc-cpVenus (aka hGcpV) protein: construct and pH-dependent BRET emission spectra. (**B**, **C**) Insensitivity of hGluc-cpVenus spectra to NaCl (**B**) and KCl (**C**) within the range of 0–300 mM NaCl or KCl. (**D**) eBAF-Y protein: construct and pH-dependent BRET emission. Sensitivity of eBAF-Y spectra to changes of NaCl (**E**) and KCl (**F**). Spectra were normalized to luminescence at 475 nm.
(TIF)
