# Supplementary figures and images for: pHlash: A New Genetically Encoded and Ratiometric Luminescence Sensor of Intracellular pH
Source: PLoS One. 2012 Aug 14;7(8):e43072. doi: 10.1371/journal.pone.0043072 (PMC3419163; doi:10.1371/journal.pone.0043072)

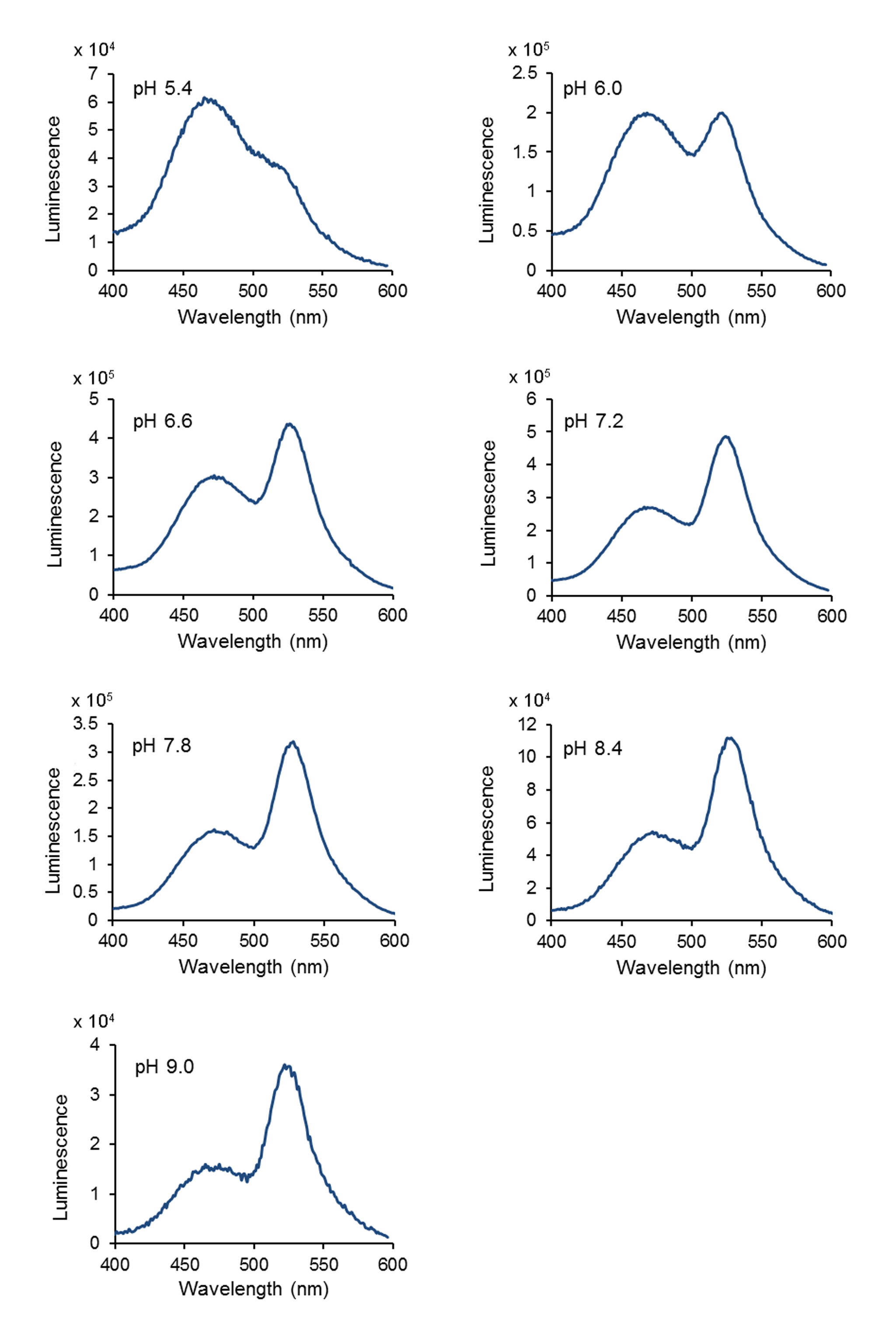

Supplement: Figure S1 — Data for some of the pH values shown in Figure 2B replotted as a single trace per pH assessment of the BRET emission spectrum. Representative pHs throughout the entire pH range are shown. Data are not normalized, note that the values of the ordinates are different among the various plots. (TIF) [file pone.0043072.s001.tif]

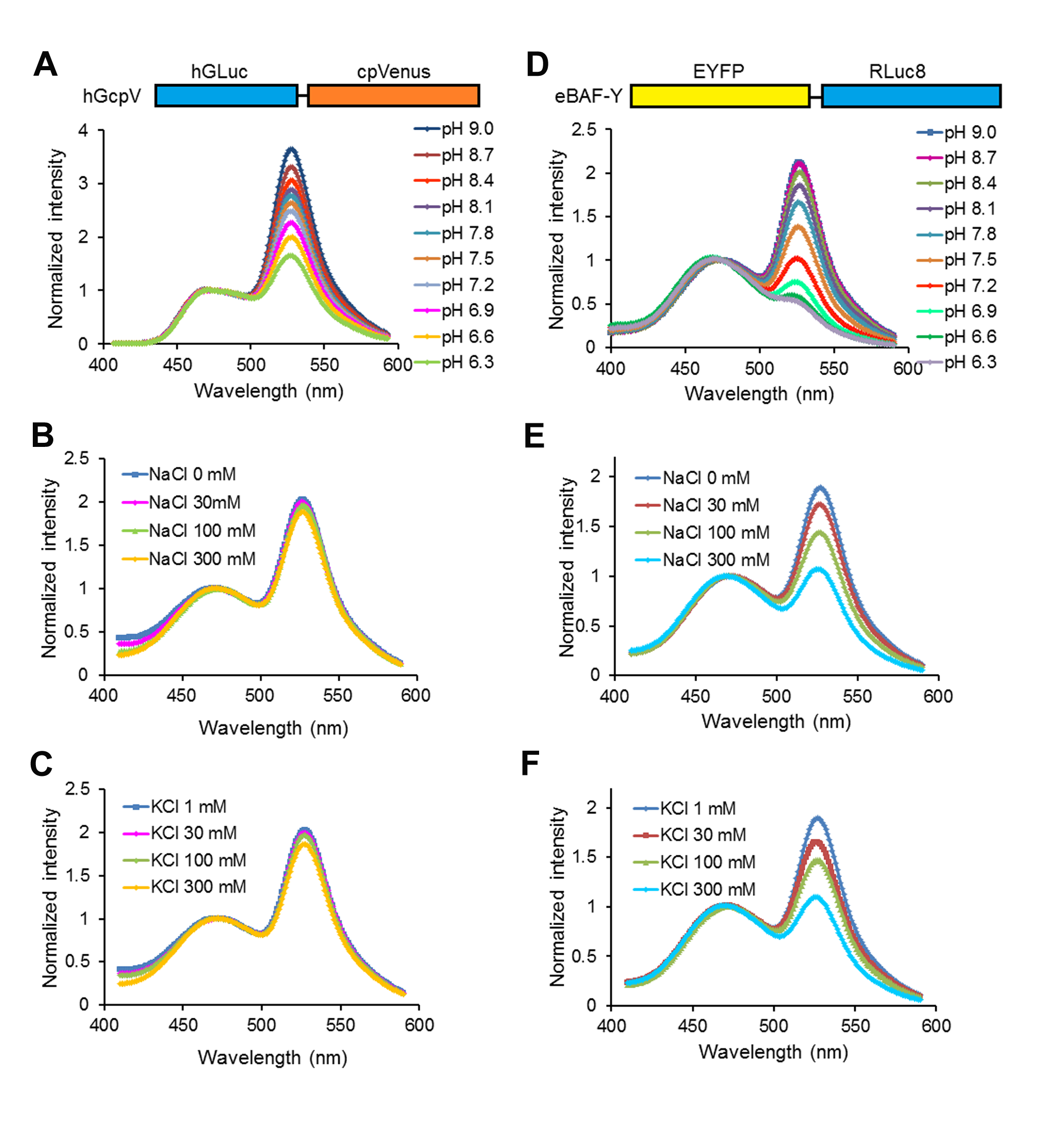

Supplement: Figure S2 — Response of purified hGluc-cpVenus and eBAF-Y to pH and NaCl/KCl. (A) purified hGluc-cpVenus (aka hGcpV) protein: construct and pH-dependent BRET emission spectra. (B, C) Insensitivity of hGluc-cpVenus spectra to NaCl (B) and KCl (C) within the range of 0–300 mM NaCl or KCl. (D) eBAF-Y protein: construct and pH-dependent BRET emission. Sensitivity of eBAF-Y spectra to changes of NaCl (E) and KCl (F). Spectra were normalized to luminescence at 475 nm. (TIF) [file pone.0043072.s002.tif]
